# Moral sensitivity and caring behavior in nursing interns: the mediating role of empathy

**DOI:** 10.3389/fpubh.2024.1360940

**Published:** 2024-03-12

**Authors:** Jin yan Chen, Hong fang Chen, Xing huan Wang, Qing zhen Zong, Zhen zhen Yao

**Affiliations:** ^1^School of Nursing, Shaanxi University of Traditional Chinese Medicine, Xianyang, China; ^2^Personnel Department, Shaanxi Provincial Hospital of Traditional Chinese Medicine, Xi’an, China; ^3^Department of Nursing, Shaanxi Provincial Hospital of Traditional Chinese Medicine, Xi’an, China

**Keywords:** nursing interns, moral sensitivity, empathy, caring behavior, the mediation effect

## Abstract

**Background:**

The main purpose of this study is to analyze the relationship between moral sensitivity, empathy, and caring behaviors and to explore the mediating effect of empathy on moral sensitivity and caring behaviors of nursing interns.

**Methods:**

A cross-sectional survey was conducted from August to September 2022 in which 261 nursing interns from two Grade 3A Hospitals in Xi’an participated. The questionnaires used in the survey include the General Information Questionnaire (GIQ), the Moral Sensitivity Questionnaire—Revised Version translated into Chinese (MSQ R-CV), the Chinese version of the Jefferson Empathy Scale (JSE), and the Chinese version of the Caring Behavior Inventory (C-CBI). The obtained data were analyzed through descriptive statistics, a one-way analysis of variance (ANOVA), and Pearson’s correlation coefficient, and the mediating effect of empathy was tested through structural equations.

**Results:**

The overall mean of moral sensitivity of nursing interns in two Grade 3A Hospitals in Xi’an is 40.84 ± 8.73, the overall mean of empathy is 100.51 ± 21.56, and the overall mean of caring behavior is (113.81 ± 21.05). Statistical analysis showed that there is a positive correlation between moral sensitivity and caring behavior of nursing interns (*r* = 0.376, *p* < 0.01), between their empathy and moral sensitivity (*r* = 0.336, *p* < 0.01), and between their empathy and caring behavior (*r* = 0.394, *p* < 0.01). The empathy of nursing interns has a mediated effect on the relationship between moral sensitivity and caring behavior. The mediated effect value was 0.14, accounting for 31.82% of the total effect.

**Conclusion:**

The moral sensitivity of nursing interns can have a direct impact on predicting the caring behavior and indirect influences their caring behaviors mediated by empathy, with the latter effect being mediated by empathy. Therefore, nursing educators and hospital administrators should adopt targeted interventions to improve the moral sensitivity and empathy of nursing interns, which can further prove to be beneficial in improving their caring behaviors, leading to enhanced quality of nursing care and reduced nurse–patient conflicts and finally to a stabilized nursing team.

## 1 Introduction

*The Outline of the Healthy China 2030 Plan* pointed out that “it is necessary to strengthen the humanistic care of medical services and to improve the humanistic caring ability of nurses” (1). Caring behavior refers to the intentional behavior of nurses paying attention to the physical care and emotional concerns of patients to improve the latter’s sense of security (2). Lack of caring behavior in nurses increases nurse–patient conflicts and leads to their burnout (3). At present, the nursing curriculum in China mainly focuses on imparting skills and pays little attention to humanities. Most courses are designed to teach only theory. In addition, a scenario of Insufficient cooperation between nursing schools and hospitals and not adding humanistic caring ability in practical education have led to nursing interns in China having a low humanistic caring ability (4). As nursing interns form the reserve force of the nursing team, their caring behavior will directly affect the quality of future clinical care.

Moral sensitivity, also known as ethical sensitivity, refers to the adherence to moral values in a conflict situation and self-awareness of one’s roles and responsibilities in that situation (5, 6). This is a prerequisite for individuals to engage in ethical decision-making and behavior (7). In China, nurses often encounter various ethical issues during their clinical work, and their improper handling causes increased psychological pressure on them or even their departure from the job (8). Currently, the total score of moral sensitivity of nursing interns is at a moderate level with some room for improvement (9). Johnson et al. showed that the higher the moral sensitivity the nurses have, the more proactive they are in providing care and assistance to patients (10). Enhancing the moral sensitivity of nursing staff could improve the quality of care and stabilize the nursing team (11). Therefore, it is clear that a high level of moral sensitivity can motivate nursing interns to provide a better quality of service to patients, which is important for the development of nursing and human health.

Empathy, also known as sympathy (12), is the ability to understand a patient’s situation, feelings, and actions from the perspective of the patient in a helpful or therapeutic way (13). Empathy, an important part of medical humanities, is the basic and practical quality that nurses should have to foster a healthy nurse–patient relationship. Empathy is seen as an important component of a supportive relationship, and the higher the level of empathy, the greater the humanistic caring skills nurses will have (14). A research survey has shown that nursing interns experience a decline in empathy when they enter the clinical practicum phase (15). In addition, research has shown that there is a positive correlation between nurses’ moral sensitivity and their empathy (16). Nurses with a high level of empathy are more able to communicate effectively with patients and their families and are more likely to think differently to identify patient needs in a timely manner and make clinical decisions that are in the interests of the patient (17).

Although previous studies have proved that nurses’ moral sensitivity, empathy, and caring behavior are positively correlated (10, 14, 16), most of them have focused on the general nurse population. There are fewer studies on the specific mechanism of the role of and on the construction of a model using these three variables. Therefore, this study focuses on a group of nursing interns to investigate the intrinsic mechanisms and mediating effects of empathy, moral sensitivity, and caring behavior among them. Based on the previous literature review, the following hypotheses about the nursing interns are formulated:

*H1*: Moral sensitivity is positively correlated with caring behaviors.

*H2*: Empathy is positively correlated with moral sensitivity.

*H3*: Empathy is positively correlated with caring behaviors.

*H4*: Empathy mediates the effect of moral sensitivity and caring behavior.

The purpose of this study is to explore the intrinsic mechanisms and mediating effects of empathy, moral sensitivity, and caring behaviors among nursing interns and further to provide a basis for nursing educators and hospital administrators to develop strategies to improve the caring behaviors of nursing interns.

## 2 Methods

### 2.1 Subjectivity and data collection

#### 2.1.1 Subjectivity

A cross-sectional survey was conducted from August to September 2022 in which 261 nursing interns from two Grade 3A Hospitals in Xi’an participated. The inclusion criteria were internship in the hospital for more than 1 month and consent to participate in this survey. The exclusion criteria included the actual internship period for shorter than 1 month for various reasons and other reasons that make them not suitable for this survey. According to descriptive research, the sample size formula is as follows (18): N = [number of variables× (5–10)×[1 + (10–15%)]. There were 19 variables in this survey, and considering 15% of the questionnaires as invalid, the final sample size was determined to be 109–219. The final sample size of this study was 261.

#### 2.1.2 Data collection

The QR code of the questionnaire was created through the Questionnaire Star Platform. After getting permission from each hospital, an uniformly trained researcher contacted the nursing department and distributed the questionnaire to the respondents through WeChat. The questionnaire was answered anonymously. To help the respondents understand the purpose of the study and know how to complete the questionnaire, guidelines were provided at the beginning of the questionnaire. Among 280 questionnaires sent out, 261 valid responses were received, accounting for the response rate of 93.21%. Moreover, 19 invalid questionnaires were excluded for being answered regularly or wrongly.

### 2.2 Measurements

#### 2.2.1 General information questionnaire

The general information questionnaire, designed by the research team, elicited the sex, age, level of education, internship period, and place of birth of the participants and featured questions such as whether you are the only child of your family, whether you have any religious beliefs, have you ever been hospitalized? whether you have a degree of fondness of your major, whether you have ever taken a nursing ethical course, and whether you have ever received any humanistic caring training during your school or training in the hospital.

#### 2.2.2 Moral sensitivity questionnaire-revised version into Chinese (MSQ R-CV)

The Moral Sensitivity Questionnaire-Revised Version into Chinese (MSQ R-CV) was translated into Chinese by Huang et al. (6). It contains two dimensions and nine items after cultural adaptation. The nine items are moral strength and responsibility (five items) and a sense of moral burden (four items). The items were rated on a 6-point Likert scale from “completely disagree” to “completely agree,” with a total score varying between 9 and 54 points. The higher the scores, the greater moral sensitivity nurses have. The Cronbach’s α coefficient of this questionnaire was 0.929.

#### 2.2.3 The Chinese version of Jefferson Empathy Scale (JSE)

The Chinese version of the Jefferson Empathy Scale (JSE), which was translated into Chinese by Ma Li (19), was mainly used for self-assessment of nursing staff’s empathy. This questionnaire had three dimensions (20 items in total), including viewpoint selection, emotional care, and transpersonal thinking, and it was scored on a 7-point Likert scale, ranging from “strongly disagree” to “strongly agree,” with 10 items scored in a reverse order. The total score of the scale was between 20 and 140, with higher scores indicating higher levels of nurses’ awareness on empathy. The Cronbach’s α coefficient of this questionnaire was 0.945.

#### 2.2.4 The Chinese version of Caring Behaviors Inventory (C-CBI)

The Chinese version of the Caring Behaviors Inventory (C-CBI) was translated into Chinese by Da Chaojin (20). This inventory included respect and connection (10 items), knowledge and skills (five items), and support and reassurance (nine items) in three dimensions (24 items in total). A 6-point Likert scale was used, ranging from “never” to “always.” The total score was in the range of 24–144. The higher the score, the better the caring behavior of the nursing interns. Based on the mean score of the items, the caring behavior was divided into three levels, with a score of <2 as the low level, 2 to <5 as the medium level, and ≥ 5 as the high level. The Cronbach’s α coefficient for this scale was 0.977.

### 2.3 Statistical analysis

Statistical analysis was performed using Excel, SPSS 26.0 software, and AMOS 24.0 software. Enumeration data are expressed as frequencies and percentages, and part of the data that conform to a normal distribution are expressed as $\bar{x}$± S. A one-way analysis of variance (ANOVA) was used to compare the scoring differences in empathy, moral sensitivity, and caring behavior among nursing interns whose demographics are different. Pearson’s correlation analysis was used to explore the correlation among empathy, moral sensitivity, and caring behaviors of the nursing interns. The mediating effect of empathy between moral sensitivity and caring behavior was tested by the Bootstrap method using AMOS 24.0 software, and the difference was considered statistically significant at a *p*-value of <0.05.

## 3 Results

### 3.1 Moral sensitivity, empathy, and caring behavior scores of nursing interns with different demographic characteristics

Table 1 shows the characteristics of the participants and the scores of moral sensitivity, empathy, and caring behaviors of nursing interns coming from different demographic backgrounds Of the 261 nursing interns, (1) 44 were male, accounting for 16.9% of the total intern population, and 217 were female, accounting for 83.1% of the total intern population (sex); (2) 65 nurses were 20 years old, accounting for 24.9%, 139 nurses were 20–25 years old, accounting for 53.3%, and 57 nurses were > 25 years old, accounting for 21.8% of the population (age); and (3) 37 nurses were college students, accounting for 14.2% of the population, 205 nurses were undergraduates, accounting for 78.5% of the population, and 19 nurses were masters, accounting for 7.3% of the population (level of education). (4) The internship period was 1–5 months for 122 nursing interns, accounting for 46.7% of them, 6–10 months for 132 nursing interns, accounting for 50.6% of them, 11–15 months for 7 nursing interns, accounting for 2.7% of them. (5) The place of birth was city for 52 nursing interns (19.9%), town for 39 of them (14.9%), countryside for 170 of the participants (65.1%). (6) To the question whether you are the only child in your family, responses from 45 were that they were the only child, accounting for 17.2%, and 216 were not the only child, accounting for 82.8%. (7) For the question whether you have any religious beliefs, eight held religious beliefs, accounting for 3.1%, and 253 had no religious beliefs, accounting for 96.9%. (8) To the question have you ever been hospitalized? 96 nursing interns responded positively, accounting for 36.8%, while 165 did not have that experience, accounting for 63.2%. (9) For the degree of fondness of your major, the responses were “very like” by 25 nursing interns, accounting for 9.6% of them; “like” by 120 nursing interns, accounting for 46.0% of them; “unclear” by 78 nursing interns, accounting for 29.9% of them; “dislike” by 30 nursing interns, accounting for 11.5% of them; and “very dislike” by eight nursing students, accounting for 3.1% of them. (10) When asked whether you have ever learned a nursing ethical course, 226 nursing students said they had learned, 18 nursing interns have not learned but knew something about it and 17 nursing interns have not learned and still knew nothing about it. (11) To the question whether you have ever received any humanistic caring training during your school or training in the hospital, 217 said yes (accounting for 83.1%), while 44 said no (16.9%). The result of the univariant analysis showed that there is a statistically significant difference (*p* < 0.05) on (1) the moral sensitivity of nursing interns in terms of sex, fondness of their major, and receiving humanistic caring training; (2) the empathy of nursing interns in terms of fondness of their major and receiving humanistic caring training; and (3) the caring behaviors of nursing interns in terms of sex, fondness of their major, learning the nursing ethical courses, and receiving humanistic caring training.

**Table 1:** Moral sensitivity, empathy and caring behavior scores of nursing interns from two Grade 3A hospitals in Xi’an City (*N* = 261).

	*N*(%)	Moral sensitivity	t/F	*p*	Empathy	t/F	*p*	Caring behavior	t/F	*p*
Sex										
Male	44(16.9%)	4.87 ± 0.81	2.67	*p* < 0.05	4.91 ± 1.16	0.609	*p* < 0.05	5.04 ± 0.82	2.359	*p* < 0.05
Female	217(83.1%)	4.44 ± 0.99			4.79 ± 1.21			4.70 ± 0.88		
Age										
20	65(24.9%)	4.60 ± 1.02	0.355	*p* > 0.05	4.93 ± 1.22	0.487	*p* > 0.05	4.77 ± 1.06	1.804	*p* > 0.05
20–25	139(53.3%)	4.48 ± 0.99			4.75 ± 1.11			4.68 ± 0.82		
>25	57(21.8%)	4.51 ± 0.87			4.80 ± 1.39			4.94 ± 0.80		
Levels of education										
College students	37(14.2%)	4.18 ± 0.92	3.049	*p* > 0.05	4.78 ± 1.24	0.306	*p* > 0.05	4.68 ± 0.82	0.194	*p* > 0.05
Undergraduate	205(78.5%)	4.55 ± 1.00			4.83 ± 1.22			4.77 ± 0.90		
Masters	19(7.3%)	4.77 ± 0.64			4.61 ± 1.02			4.82 ± 0.81		
Internship period										
1–5 months	122(46.7%)	4.53 ± 0.89	0.431	*p* > 0.05	4.73 ± 1.17	0.565	*p* > 0.05	4.67 ± 0.88	1.383	*p* > 0.05
6–10 months	132(50.6%)	4.49 ± 1.06			4.89 ± 1.24			4.83 ± 0.88		
11–15 months	7(2.7%)	4.84 ± 0.79			4.69 ± 0.96			5.00 ± 0.81		
Place of birth										
City	52(19.9%)	4.58 ± 1.08	0.195	*p* > 0.05	4.77 ± 1.36	0.072	*p* > 0.05	4.84 ± 1.04	0.35	*p* > 0.05
Town	39(14.9%)	4.54 ± 1.13			4.87 ± 1.12			4.69 ± 0.71		
Countryside	170(65.1%)	4.49 ± 0.90			4.80 ± 1.18			4.75 ± 0.86		
Only child or not?										
Yes	45(17.2%)	4.40 ± 1.02	−0.919	*p* > 0.05	4.71 ± 1.31	−0.578	*p* > 0.05	4.87 ± 1.00	0.924	*p* > 0.05
No	216(82.8%)	4.54 ± 0.97			4.83 ± 1.18			4.73 ± 0.85		
Having religious beliefs?										
Yes	8(3.1%)	4.78 ± 1.02	0.789	*p* > 0.05	4.36 ± 1.03	−1.074	*p* > 0.05	4.90 ± 1.03	0.455	*p* > 0.05
No	253(96.9%)	4.51 ± 0.97			4.82 ± 1.21			4.75 ± 0.88		
Had in hospital?										
Yes	96(36.8%)	4.49 ± 1.03	−0.326	*p* > 0.05	4.81 ± 1.21	0.028	*p* > 0.05	4.84 ± 0.88	1.138	*p* > 0.05
No	165(63.2%)	4.53 ± 0.95			4.81 ± 1.20			4.71 ± 0.88		
Degree of fondness of the major										
Very like	25(9.6%)	5.21 ± 0.90	4.558	*p* < 0.05	5.55 ± 1.16	2.783	*p* < 0.05	5.12 ± 0.75	2.703	*p* < 0.05
Like	120(46.0%)	4.48 ± 0.95			4.74 ± 1.16			4.80 ± 0.86		
Unclear	78(29.9%)	4.51 ± 0.85			4.69 ± 1.07			4.75 ± 0.84		
Dislike	30(11.5%)	4.16 ± 1.09			4.73 ± 1.59			4.39 ± 1.07		
Very dislike	8(3.1%)	4.28 ± 1.40			4.93 ± 1.04			4.48 ± 0.81		
Have you ever taken a nursing ethical course?										
Yes	226(86.6%)	4.55 ± 0.97	2.201	*p* > 0.05	4.86 ± 1.16	1.909	*p* > 0.05	4.81 ± 0.86	3.054	*p* < 0.05
Never and knew nothing about it	17(6.5%)	4.04 ± 1.09			4.32 ± 1.61			4.33 ± 1.14		
Never, but knew some about it	18(6.9%)	4.59 ± 0.79			4.60 ± 1.20			4.52 ± 0.74		
Have received any humanistic caring training?										
Yes	217(83.1%)	4.59 ± 0.94	2.621	*p* < 0.05	4.87 ± 1.16	1.979	*p* < 0.05	4.81 ± 0.85	2.269	*p* < 0.05
No	44(16.9%)	4.17 ± 1.06			4.48 ± 1.36			4.49 ± 1.00		

### 3.2 Current situation of moral sensitivity, empathy, and caring behaviors of nursing interns

Table 2 shows the scores of moral sensitivity, empathy, and caring behavior of the participating nursing interns. The overall mean scores of moral sensitivity, empathy, and caring behavior are 40.84 ± 8.73, 100.51 ± 21.56, and 113.81 ± 21.05, respectively.

**Table 2:** Moral sensitivity, empathy, and caring behavior scores of nursing interns (*N* = 261, $\bar{x}$ ± S).

Variables	Items	Scoring range	Mean ± SD	Mean ± SD
Moral sensitivity	9	9 ~ 54	40.84 ± 8.73	4.52 ± 0.97
Moral strength and responsibility	5	5 ~ 30	23.56 ± 5.16	4.71 ± 1.03
Sense of moral burden	4	4 ~ 24	17.29 ± 4.39	4.32 ± 1.10
Empathy	20	20 ~ 140	100.51 ± 21.56	4.81 ± 1.20
Viewpoint selection	10	10 ~ 70	55.96 ± 9.61	5.60 ± 0.96
Emotional care	8	8 ~ 56	35.87 ± 11.49	4.49 ± 1.44
Transpersonal thinking	2	2 ~ 14	8.68 ± 3.46	4.34 ± 1.74
Caring behavior	24	24 ~ 144	113.81 ± 21.05	4.76 ± 0.88
Respect and connection	10	10 ~ 60	46.06 ± 9.66	4.61 ± 0.97
Knowledge and skills	5	5 ~ 30	24.07 ± 4.90	4.81 ± 0.98
Support and reassurance	9	9 ~ 54	43.68 ± 8.58	4.85 ± 0.95

### 3.3 Correlation analysis of moral sensitivity, empathy, and caring behavior of nursing students

Table 3 shows the results of the correlation analysis of moral sensitivity, empathy, and caring behavior of nursing interns. There exists a positive correlation between all dimensions of moral sensitivity and all dimensions of caring behaviors (*r* = 0.376, *p* < 0.01), between all dimensions of empathy and all dimensions of moral sensitivity (*r* = 0.336, p < 0.01), and between all dimensions of empathy and caring behaviors (*r* = 0.394, *p* < 0.01).

**Table 3:** Correlation analysis of moral sensitivity, empathy, and caring behaviors of nursing interns (*N* = 261).

Variables	1	2	3	4	5	6	7	8	9	10	11
1	1.000										
2	0.908*	1.000									
3	0.919*	0.670*	1.000								
4	0.336*	0.300*	0.314*	1.000							
5	0.216*	0.203*	0.192*	0.809*	1.000						
6	0.230*	0.189*	0.230*	0.875*	0.614*	1.000					
7	0.389*	0.355*	0.356*	0.905*	0.618*	0.650*	1.000				
8	0.376*	0.366*	0.322*	0.394*	0.319*	0.270*	0.418*	1.000			
9	0.367*	0.340*	0.330*	0.387*	0.278*	0.280*	0.418*	0.894*	1.000		
10	0.343*	0.335*	0.294*	0.356*	0.303*	0.244*	0.370*	0.922*	0.729*	1.000	
11	0.314*	0.322*	0.255*	0.331*	0.288*	0.212*	0.353*	0.912*	0.710*	0.783*	1.000

*p < 0.01, 1 = Moral sensitivity; 2 = moral strength and responsibility;3 = sense of moral burden; 4 = empathy; 5 = viewpoint selection; 6 = emotional care; 7 = transpersonal thinking; 8 = caring behavior; 9 = respect and connection; 10 = knowledge and skills; 11 = support and reassurance.

### 3.4 Analysis of the mediating effect of empathy between moral sensitivity and caring behavior

**Figure 1:**
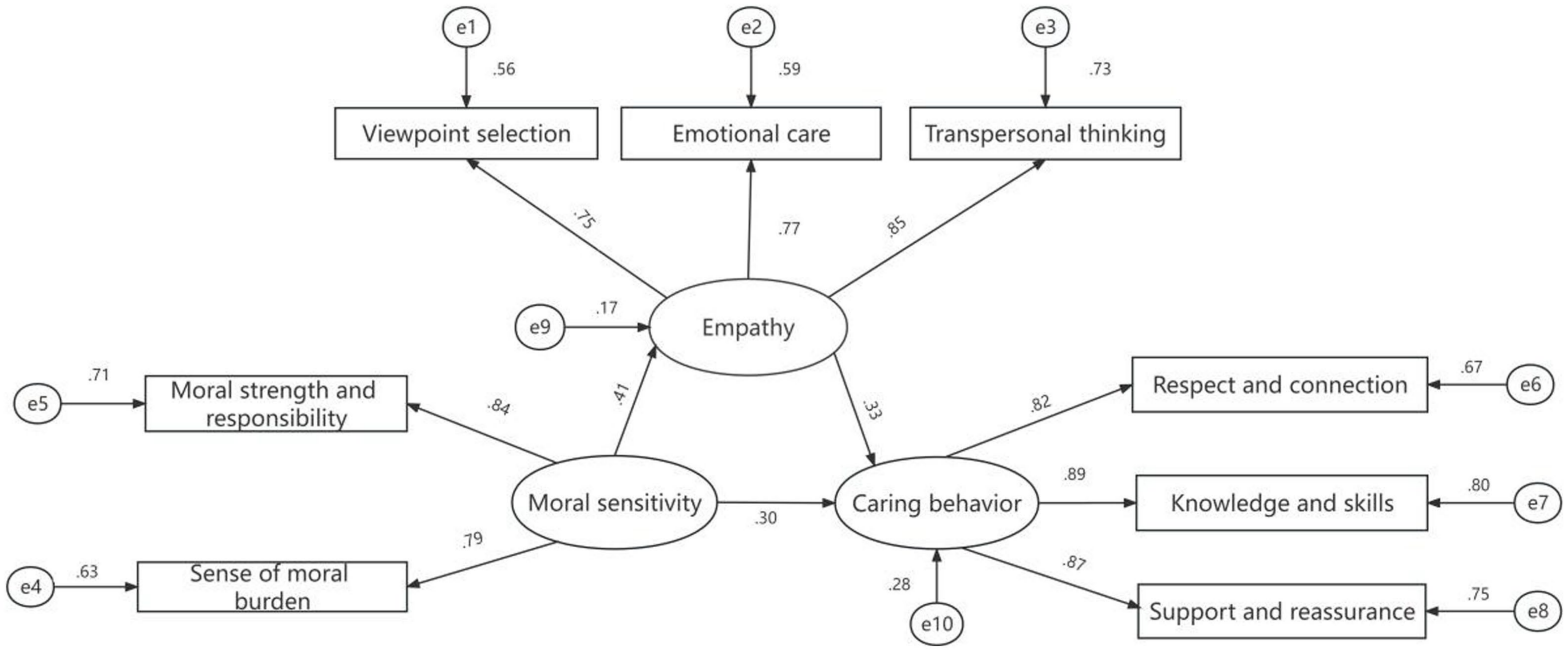
Structural equation model of the mediating effect of empathy between moral sensitivity and caring behaviors among 261 nursing interns in 2 Grade 3A hospitals in Xi’an City, China.

To further validate the mechanism of moral sensitivity on caring behavior, a structural equation model was developed with moral sensitivity as the independent variable, caring behavior as the dependent variable, and empathy as the mediating variable. The mechanism was validated using AMOS 24.0 software (see Figure 1). The results of model fitting showed that χ2/df = 1.799, RMSEA = 0.055, GFI = 0.97, AGFI = 0.937, NFI = 0.971, RFI = 0.952, IFI = 0.987, TLI = 0.978, and CFI = 0.987 and all the fitting indexes were located at the standard range (see Table 4). The estimation results of the model path coefficients show that all factor loadings are *p* < 0.01 (see Table 5). After testing through the Bootstrap procedure with 2000 repetitions of sampling and calculation on 95% CI, the results showed that the 95% CI for the direct and indirect effects of moral sensitivity on caring behaviors of nursing interns did not contain zero, suggesting that empathy partially mediates the relationship between moral sensitivity and caring behaviors. The direct effect value of nursing interns’ moral sensitivity on caring behavior was 0.30, the mediating effect value was 0.14 (0.41 × 0.33), the total effect value was 0.44 (0.30 + 0.14), and the mediating effect percentage was (0.14/0.44) × 100% = 31.82% (see Table 6).

**Table 4:** Structural equation model fitting metrics.

Fit indices	χ^2^/df	RMSEA	AGFI	GFI	NFI	CFI	RFI	IFI	TLI
Result	1.799	0.055	0.937	0.97	0.971	0.987	0.952	0.987	0.978
Threshold	<3	<0.08	>0.9	>0.9	>0.9	>0.9	>0.9	>0.9	>0.9

**Table 5:** Estimation results of model path coefficients.

Path	Estimate	SE	CR	*p*
Empathy<−--Moral sensitivity	0.342	0.065	5.219	*p* < 0.01
Caring behavior<−--Empathy	0.365	0.084	4.359	*p* < 0.01
Caring behavio<−--Moral sensitivity	0.276	0.07	3.949	*p* < 0.01

**Table 6:** Effect values of moral sensitivity, empathy, and caring behavior of nursing interns in two Grade 3A hospitals in Xi’an, China.

Effect	Path	Standardized effect value	Percent (%)	95%*CI*
Direct effect	Moral sensitivity→Caring behavior	0.30	68.18%	0.120 ~ 0.480
Indirect effect	Moral sensitivity→Empathy→Caring behavior	0.14	31.82%	0.068 ~ 0.227
Total effect		0.44	100%	0.264 ~ 0.590

## 4 Discussion

### 4.1 Analysis on the current situation of nursing interns’ moral sensitivity, empathy, and caring behavior

At first, the results of this study showed that the nursing interns’ scores on moral sensitivity was 40.84 ± 8.73, which was at a medium level and was lower than the results of the study by Ouyang Lixia (21). The reason for the medium score on moral sensitivity have been explained in previous studies. The nursing age is an influencing factor on the moral sensitivity of the nurses, and the higher the nursing age, the richer clinical experiences the nurses have. When facing ethical issues, the nurses with richer experiences have the ability to adopt a skillful and calm approach to solve the problem (22). The nursing interns, experiencing a period of transition from campus to clinical work, are unfamiliar with nursing skills and lack clinical experience and ethical practice. Therefore, it is recommended that schools and hospital administrators help them understand and analyze the ethical clinical issues and improve their moral sensitivity through game-based teaching methods, debate-based teaching methods, lectures on ethics, role-playing, and the creation of a positive ethical environment (23–25). In addition, Palazoğlu et al. (26) showed that nurses’ moral sensitivity can be improved through training and education, suggesting that nursing administrators can improve the moral sensitivity of nursing interns by reinforcing this content in their daily training.

Then, the results in this study showed that the nursing interns’ scores on empathy was 100.51 ± 21.56 at a medium level and slightly lower than the results of Zhao Y (27) and other authors, which can be explained through the fact that, compared with nursing interns, nurses in military hospital received psychological education more widely, which helps them more easily accept the feelings and ideas of the patients. The level of empathy could be motivated, cultivated, and improved by repetitive practice, imaginative teaching methods, good examples, and learning under teachers’ supervision (28). Therefore, schools need to include empathy-related courses in their curriculum to cultivate nursing interns’ levels of empathy and the hospital administrators should strengthen and promote empathy-related education through setting good examples. In addition, studies have shown that it is important to focus on the psychological adjustment of nurses and that empathy training can be carried out by means of narrative nursing education (29) and digital storytelling (30) in order to enhance the level of empathy of nursing interns.

Next, the results of this study showed that the nursing interns’ scores on caring behavior was 113.81 ± 21.05, which was at a medium level. These scores were higher than the normative score of clinical nurses in China (77.0 ± 17.36) (31) and lower than the results reported by Zhang ZQ (32) and others. This finding may be related to later development of nursing education in China. The single form of teaching focuses on fundamental courses and does not include humanistic caring courses and related training (33). Therefore, schools need to offer more humanistic caring courses. Clinical instructors need to establish a harmonious and stable nurse–patient relationship and strengthen the use of nurse–patient communication skills in the clinic, so as to enhance the nursing nurses’ understanding of the importance of caring behaviors and to improve their caring behaviors. In addition, caring behaviors of nursing interns can be enhanced through training such as role modeling, cognitive reappraisal training, non-violent communication (34), emotion regulation skills training (35), and humanistic care lectures.

### 4.2 Correlation analysis on nursing interns’ moral sensitivity, empathy and caring behaviors

The results of this study also showed that moral sensitivity and caring behaviors of nursing interns were positively correlated, validating Hypothesis H1, namely, as moral sensitivity increases, caring behaviors increase, and conversely moral insensitivity or inability to recognize ethical challenges may lead to inappropriate caring behaviors, which is in line with the findings of the literature (36, 37). Nursing interns with high moral sensitivity can keenly identify ethical issues, make correct ethical decisions, improve their sense of professional identity and job satisfaction, and provide quality humanistic nursing services to patients, thus enhancing the level of care for patients. Therefore, nursing educators and administrators can simulate real-life situations and adopt experiential teaching and other methods to enhance the moral sensitivity of nursing interns to improve their caring behaviors toward patients (38). The positive correlation between empathy and moral sensitivity of nursing interns verified hypothesis H2, that is, the higher the empathy, the higher the moral sensitivity, which is consistent with the findings of the literature (39, 40). Empathy, which improves moral sensibility, is one of the most important aspects of nursing ethics, without which, nursing interns could not appropriately identify the needs and problems of their patients and fulfill their ethical responsibilities. Therefore, nursing educators and hospital administrators need to design a systematic and practical training program for improving the levels of empathy and moral sensibility among nursing students. The positive correlation between empathy and caring behaviors of nursing interns verified hypothesis H3—improving the empathy skills of nursing interns would improve their caring behaviors toward patients, which is similar to the findings of the literature (38, 41). Nursing interns with higher levels of empathy were able to put themselves in the patient’s shoes, understand the patient’s sorrow and unhappiness in a timely manner, and provide them with appropriate help and support, which in turn resulted in an increased care for the patients. Therefore, nursing educators and hospital administrators need to enhance the caring competence of nursing interns through group counseling on empathy, clinical simulation teaching, and the formation of a systematic training program on empathy competence.

### 4.3 Analysis on the mediating effect of empathy between moral sensitivity and caring behavior in nursing interns

Finally, the results of this study showed that the empathy of nursing interns partially mediated the relationship between moral sensitivity and caring behavior, with a mediating effect value of 0.14, accounting for 31.82% of the total effect, which suggests that the moral sensitivity of nursing interns directly predicts their caring behavior and indirectly influences their caring behavior mediated by empathy, validating hypothesis H4. Nursing interns with high empathy have high moral sensitivity and can promptly understand the sorrow and needs of patients, think from a transpersonal perspective, focus on communication with patients, empathize with patients, and further display more caring behaviors during their clinical practice, such as respecting, supporting, caring, and helping (42, 43). The nursing interns with low empathy generally can neither understand the patients’ point of view nor have the ability to think about the latter’s problems. It is not easy to perceive the patients’ emotions sensitively and find out the ethical problems in time. These interns are likely to have ethical conflicts with the patients, which is not conducive to the implementation of caring behaviors. Therefore, nursing educators and hospital administrators need to focus on the improvement of nursing interns’ moral sensibility and training on their empathy, which further could promote their caring behavior, reduce the nurse–patient conflicts, and stabilize the nursing team.

### 4.4 Limitations

This study has limitations such as the small size of the sample and the restricted scope for sampling. In addition, the results of this study does not depict the overall situation of nursing interns in China because of regional differences. In future studies, the sampling range could be expanded and the relationship among moral sensitivity, empathy, and caring behavior of nursing interns in different regions and levels of hospitals could be further explored. In addition, future studies need to focus on other variables that may influence the caring behaviors of nursing interns.

## 5 Conclusion

In summary, moral sensitivity has a direct and indirect predictive effect on caring behavior, and empathy plays a partially mediating role on moral sensitivity and caring behavior. Nursing educators and hospital administrators should pay attention to the interrelationships and roles of empathy, moral sensitivity, and caring behavior and should use a variety of educational and training methods to cultivate the moral sensitivity and empathy of nursing interns so as to improve their caring behavior.

## Data availability statement

The original contributions presented in the study are included in the article/supplementary material, further inquiries can be directed to the corresponding author(s).

## Ethics statement

The studies involving humans were approved by the Ethics Committee of Shaanxi Provincial Hospital of Traditional Chinese Medicine. The studies were conducted in accordance with the local legislation and institutional requirements. The participants provided their written informed consent to participate in this study. Written informed consent was obtained from the individual(s) for the publication of any potentially identifiable images or data included in this article.

## Author contributions

JC: Conceptualization, Data curation, Formal analysis, Investigation, Methodology, Project administration, Visualization, Writing – original draft. HC: Funding acquisition, Investigation, Methodology, Project administration, Resources, Writing – review & editing. XW: Conceptualization, Data curation, Formal analysis, Investigation, Methodology, Writing – original draft. QZ: Data curation, Methodology, Project administration, Software, Writing – review & editing. ZY: Investigation, Software, Validation, Writing – original draft.
